# Yellow fever in Africa and the Americas: a historical and epidemiological perspective

**DOI:** 10.1186/s40409-018-0162-y

**Published:** 2018-08-25

**Authors:** Jean-Philippe Chippaux, Alain Chippaux

**Affiliations:** 1 UMR216, Mother and child facing tropical diseases, PRES Sorbonne Paris Cité, Université Paris Descartes, Faculté de Pharmacie, Paris, France; 2 Centre de Recherche Translationnelle, Institut Pasteur, 28 rue du Dr Roux, 75015 Paris, France; 3 Société de Pathologie Exotique, Hôpital Salpêtrière, BP50082, 75622 Paris cedex 13; 18 rue Princesse, 75006 Paris, France

**Keywords:** Yellow fever, *Aedes aegypti*, *Haemagogus* sp.*, Sabethes* sp. Vector, Arbovirus, Epidemiology, Brazil, Latin America, Africa

## Abstract

Yellow fever was transported during the slave trade in the 15th and 16th centuries from Africa to the Americas where the virus encountered favorable ecological conditions that allowed creation of a sustainable sylvatic cycle. Despite effective vector control and immunization programs for nearly a century, yellow fever epidemics reemerged in many Latin American countries, particularly Brazil. The emergence or reemergence of vector-borne diseases encompasses many intricate factors. Yellow fever outbreaks occur if at least three conditions are fulfilled: the introduction of the virus into a non-immune human community, presence of competent and anthropophilic vectors and insufficiency of prevention and/or adequate management of the growing outbreak. On the other hand, two weapons are available to constrain yellow fever: vector control and immunization. In contrast, yellow fever is absent from Asia and the Pacific despite the presence of the vector and the susceptibility of human populations to the virus. Based on a review of the global history of yellow fever and its epidemiology, the authors deliver some recommendations for improving the prevention of epidemics.

## Background

**Fig. 1:**
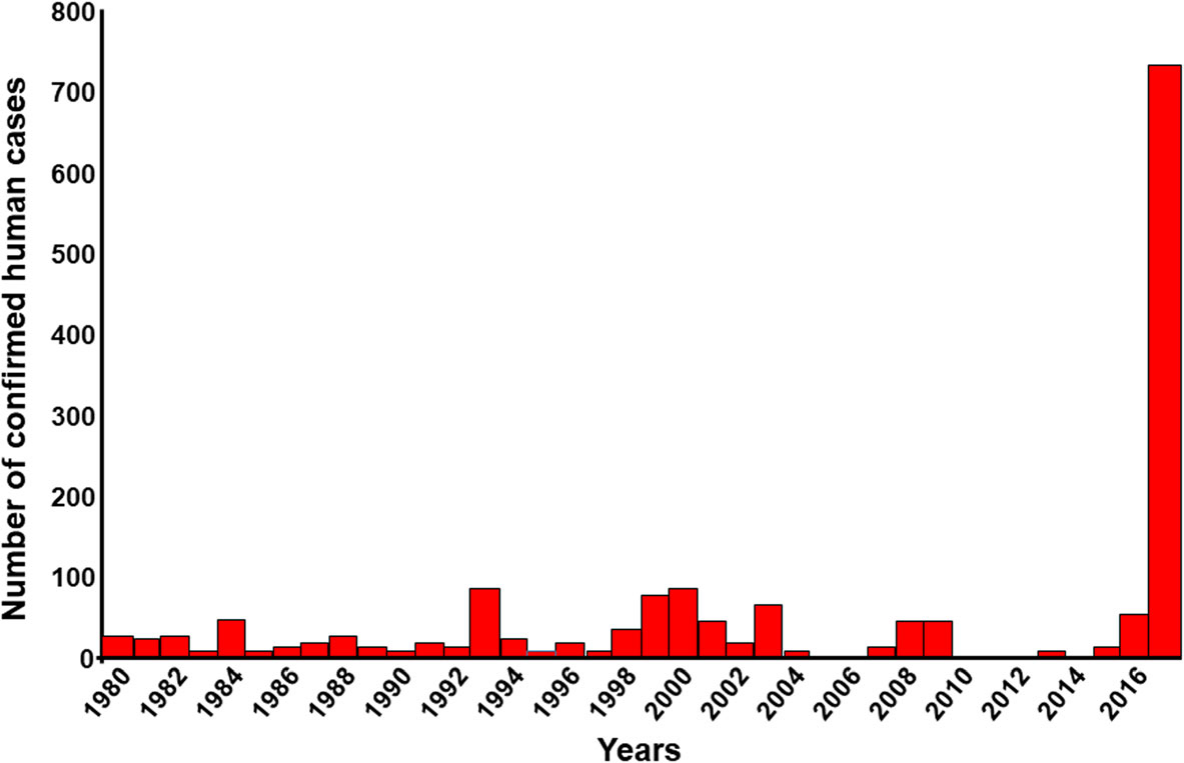
Confirmed human yellow fever cases in Brazil between 1980 and 2017 (from Sinan; GT-Arbo/UVTV/CGDT/DEVIT/SVS/MS [2])

**Fig. 2:**
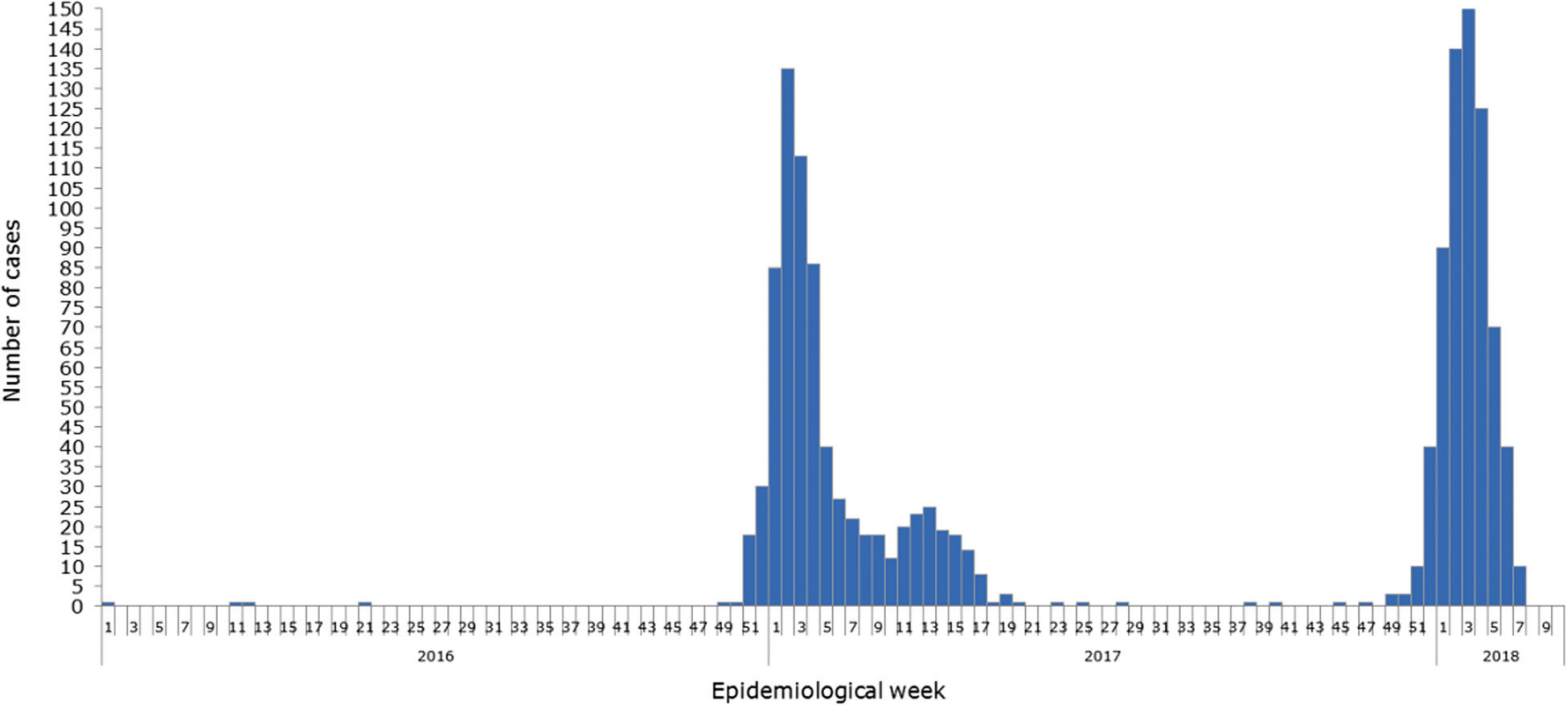
Number of confirmed yellow fever cases by epidemiological week (EW) based on date of symptom onset. Brazil, EW 1 of 2016 to EW 8 of 2018 (Source: Data published by Brazil health authorities and estimated and reproduced by PAHO/WHO: http://www.who.int/csr/don/09-march-2018-yellow-fever-brazil/en/; accessed 14/05/2018)

Brazil has experienced an exceptional yellow fever (YF) outbreak since December 2016 (Table 1). After the last major epidemic (1935–1940), sporadic cases were regularly reported from endemic states – mainly the Amazonian states – with some incursions into those of the Southeast (Minas Gerais and São Paulo, respectively in 2002 and 2008) and South (Paraná and Rio Grande do Sul in 2008), until today (Fig. 1). Preceded by an upsurge of epizootics in monkeys since 2014 [1], the current epidemic resulted, from 1 December 2016 to 8 May 2018, in 2050 confirmed cases of yellow fever including 681 deaths, indicating a case fatality rate of 33.2% (Fig. 2), while a further 1300 cases are still under investigation [2]. Despite a rapid and appropriate response, the epidemic spread to the east and south of the country, including areas generally considered non-endemic. This extension reproduces almost identically the course of all the epidemics observed in Brazil, including those following the reappearance of *Aedes aegypti* in the 1970s, after an attempt to eliminate the vector [3, 4]. However, in Brazil, this epidemic and the previous ones are marked by the role of wild vectors, *Haemagogus* sp. and *Sabethes* sp., involved in the sylvatic cycle, while *Aedes aegypti* and *A. albopictus*, which induce epidemics in urban and peri-urban areas, respectively [3, 4], do not seem to be involved in the transmission of the virus at this stage [2].

**Table 1:** Confirmed yellow fever cases and deaths in Brazil from December, 2016 to 8 May, 2018 (from [2, 131])

States	Confirmed YF cases	Deaths from confirmed YF
Pará	4	4
Tocantins	1	0
Goiás	1	1
Minas Gerais	1004	341
Espirito Santo	264	86
São Paulo	537	170
Rio de Janeiro	238	78
Distrito Federal	1	1
Total	2050	681

**Fig. 3:**
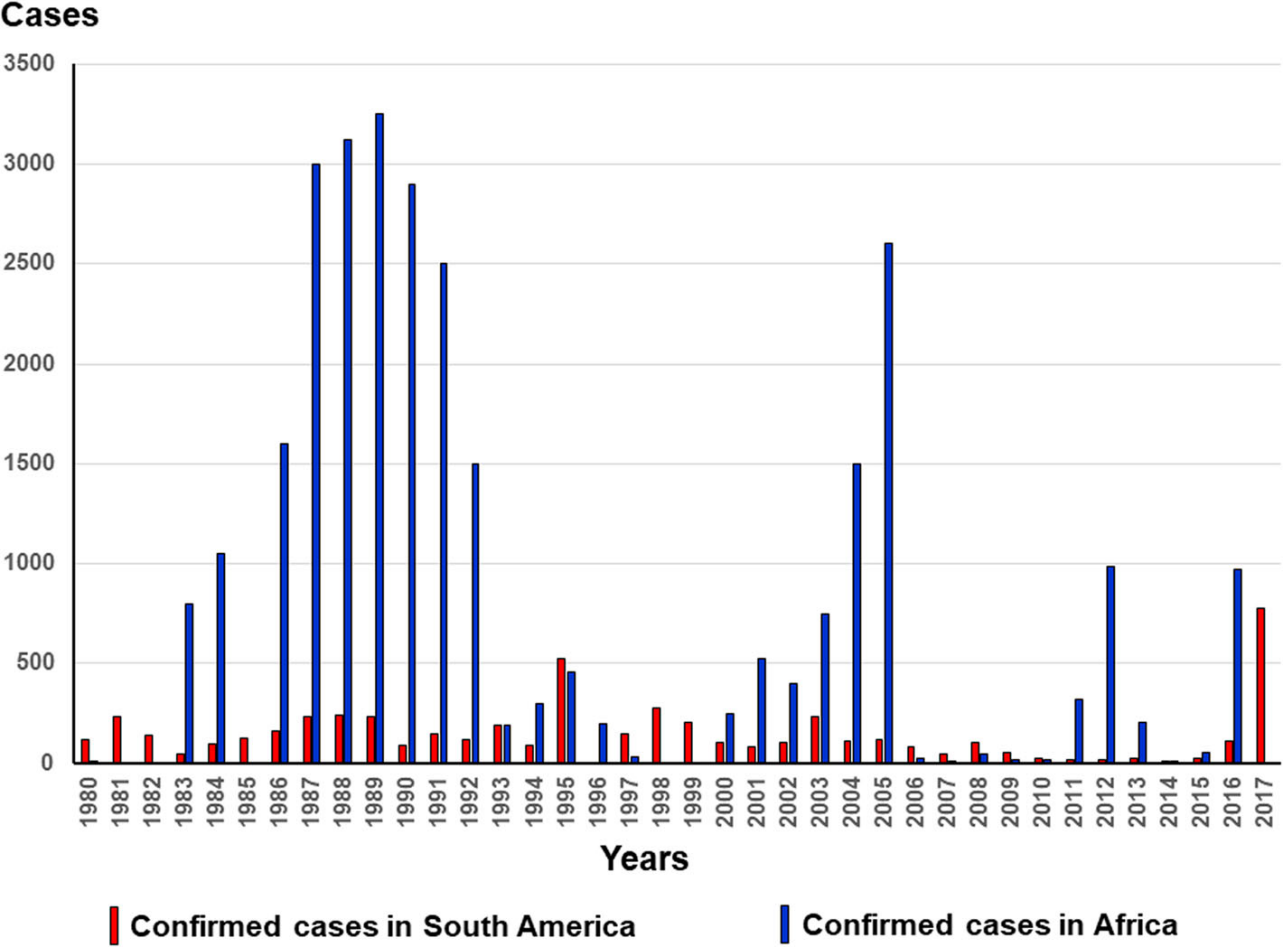
Confirmed case reports in Africa and South America between 1980 and 2017 (based on WHO, weekly epidemiological record: http://www.who.int/wer/en/ – accessed on May 18, 2018)

YF is an acute hemorrhagic hepatonephritis caused by an enveloped single-stranded RNA virus of approximately 12,000 base pairs belonging to the family Flavoviridae. It is transmitted by the bite of a mosquito belonging to the genus *Aedes* (in Africa and the Americas) or to the genera *Haemagogus* and *Sabethes* (in America). Epidemics are more frequent and important in Africa than in the Americas (Fig. 3) [5].

YF is native to Africa where it may have emerged around 3000 years ago [6]. It probably was introduced into the Americas during the beginning of the slave trade, and benefited from favorable ecological conditions, including the presence of sylvatic vectors which, although competent, forced the virus to adapt by modifying its genome to become a pathogen close to but distinct from that of Africa [7, 8].

The main objective of this review is to describe the epidemiological cycles of YF in Africa and South America, particularly Brazil, and their differences. The history of YF on the two continents, and its absence from Asia, are elements that establish the conditions necessary for its maintenance and development. Recent epidemiological studies seem to indicate characteristics that might be employed to anticipate the emergence of epidemics in major Brazilian cities and improve preventive measures.

### History of yellow fever and origin of the virus

YF probably impacted the history – and the economy – of Latin America more than those of Africa. Strongly linked to the development of the Americas, from its discovery by Europeans until the implementation of effective control strategies in the middle of the twentieth century, YF gave rise to strong social and political repercussions due, in particular, to the many deadly urban epidemics [9, 10]. It is certainly the recurrent YF epidemics that dissuaded Napoleon Bonaparte from achieving the conquest of the United States of America that he was preparing from then French Louisiana, with troops he had massed in the West Indies [11]. We can also evoke the scandal of the Panama Canal, the construction of which was delayed because of an epidemic of YF [12, 13]. In addition, research on YF has led to conflicts between scientists because of their personal pride as well as colonialist and nationalist positions [14].

The history of YF entails three main controversies – now resolved – namely its mode of transmission, geographical origin and the infectious agent responsible for the disease.

The first controversy concerned the transmission of the disease and was debated throughout the nineteenth century. The theory of contagion has long prevailed [12–14]. Like the transmission of cholera, proponents of this hypothesis defended, from the seventeenth century until the end of the nineteenth century, a transmission by water and/or human contacts, then by “miasmas”. This theory – also named “aerism” – argued that the germ penetrated into the body via the respiratory system [15]. Changes in the concepts took place gradually between the end of the eighteenth century – after the epidemics of Philadelphia in 1793, Cadiz in 1800 and Barcelona in 1821–1822, where the lack of direct contact between the patients excluded direct contamination between people – and the demonstration of the vectorial transmission in 1900 [15–17]. The “non-contagion” was first demonstrated by several doctors in the French West Indies, notably masterfully by Lefort and his team [18]. It was on this basis that Beauperthuy, as early as 1854, suggested the transmission of YF by mosquito, which he illustrated by protecting healthy persons with a bed net [19–21]. Following this new paradigm, Chervin proposed to abolish quarantining as the regular method for the prevention of YF [16]. Based on the filarial transmission model described by Manson in 1878, but without quoting the latter nor Beauperthuy, although he certainly had access to their works [14], Finlay [22] supported the vectorial transmission of the YF that was confirmed experimentally by Reed and his team in 1900–1902 [23–25]. Thus, three nations claim – with varying degrees of insistence – the discovery of the vectorial transmission of YF: France, Cuba and the USA in chronological order. The merit of the American Commission, led by Reed, was having shown that the mosquito becomes infected during the first 3 days of the disease, when the viremia is sufficiently high, and that the sting contaminating the healthy subject should occur at least 12 days later to allow replication of the virus in the mosquito [26], which legitimized the authorship of the discovery [14].

The geographical origin of YF has also long been debated. The disease was considered to have originated from the Americas, discovered at the end of the fifteenth century by the first Spanish conquerors [12]. The first description in the New World rather than in the Old one resulted from circumstantial reasons: the Americas, actual colonies for settlements and economic exploitation, attracted much more consideration than Africa, which still consisted of trading posts essentially devoted to acquiring slaves contributing to the development of the Americas. Thus, many epidemics were reported in America and the West Indies from the middle of the seventeenth century. The mention of possible or probable cases before 1647, the date of the Guadeloupe epidemic, which is generally considered the first formally identified YF epidemic in history, led to the belief that YF was already present in America at the arrival of Spanish invaders. In fact, YF would have appeared for the first time in the West Indies 2 months after the battle of La Vega-Real that Christopher Columbus launched against the Amerindians on March 24, 1495, in Hispaniola, today known as the Dominican Republic [27]. However, the disease is mentioned under multiple names based on the very recognizable symptoms, by European navigators sailing along the African coast and the Canary Islands as early as 1494, regardless of the discovery of America [12]. It is therefore likely that the first American cases resulted from an introduction of the YF virus by the crews of Columbus between 1492 and 1495 coming from the Canary Islands where the ships of Columbus made their last resupplying before the crossing of the Atlantic Ocean.

The arguments in favor of the African origin of YF prevail and this thesis has become the consensus. The frequency of epidemics and the adaptation of the virus to its hosts and vectors argue for an older presence in Africa, which molecular biology today confirms by the greater genetic heterogeneity of the virus in Africa [7, 28–31].

**Fig. 4:**
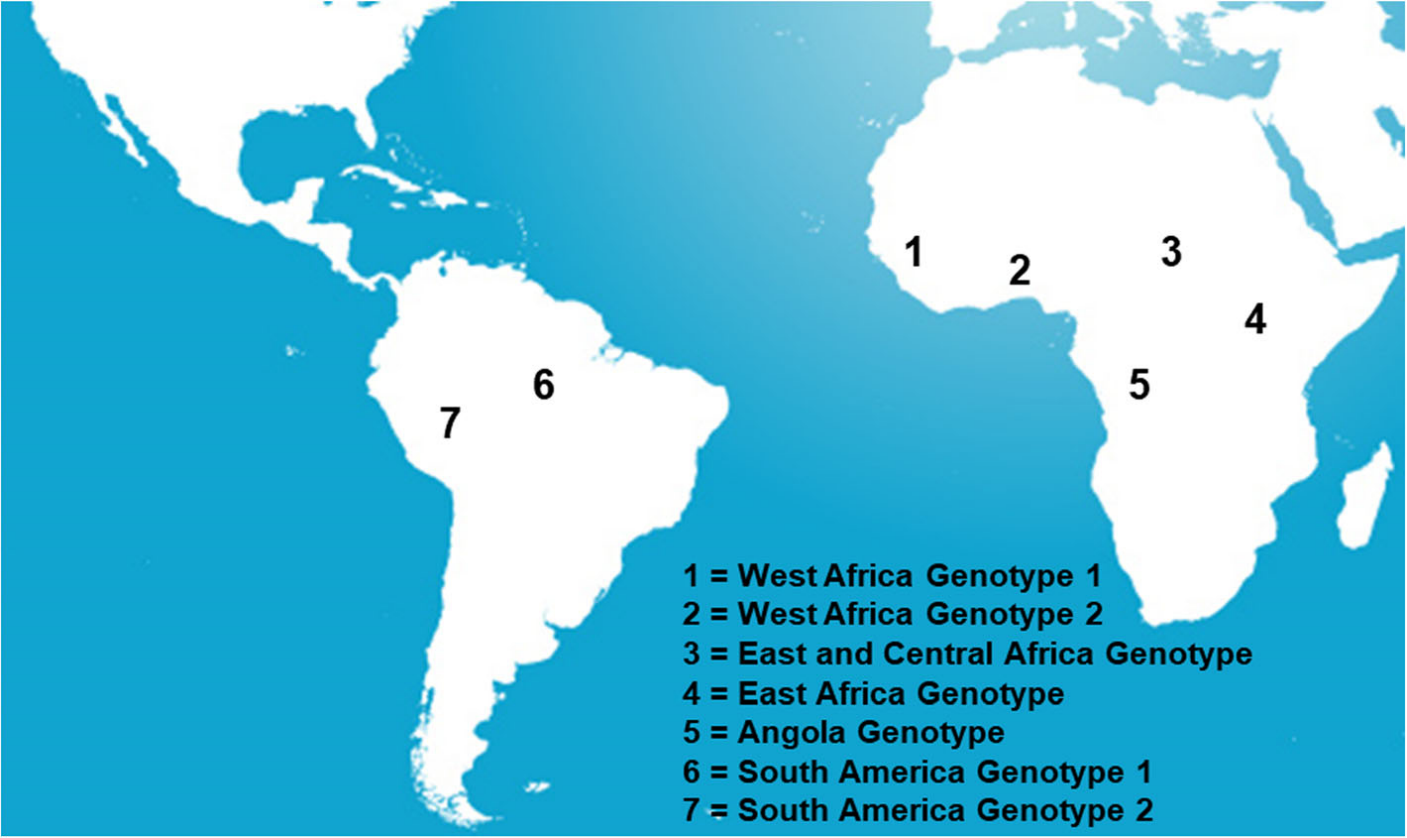
Yellow fever genotypes (adapted from [30, 31, 85–87, 89, 113])

Currently, there are seven genotypes: five in Africa and two in the Americas (Fig. 4). African genotypes are characterized by their affinity for their respective vectors [32, 33]. All the studies confirmed the African origin of the YF virus. The East African strain is the oldest, and probably diverged from an ancestral flavivirus about 3500 years ago [6, 34]. West African strains were separated from East African ones about 3 centuries before the alleged introduction of the virus into the Americas. American strains are closer to West African strains than the latter are related to the East African ones [6, 7, 34]. The virus has encountered in the Americas a competent mosquito that allowed permanent installment of a sylvatic cycle of the virus.

However, this does not explain why the disease was never mentioned in Europe before the fifteenth century, despite numerous and intense contacts between sub-Saharan Africa – the land of YF’s origin – and Europe well before the discovery of America [12]. The most likely hypothesis is the absence of a competent mosquito capable of establishing itself in a temperate zone. Subsequently, YF has been recurrent in Europe, as in North America, mainly during summer port epidemics, which are still not maintained in situ from 1 year to the next [27]. A greater number of epidemics in the Americas from the 17th to the nineteenth century in comparison with Africa resulted from a combination of socioeconomic, demographic and ecological circumstances that favored the development of the disease in relation to Africa, where many human infections were probably limited by low population density and did not result in “conspicuous” epidemics.

While an African origin of YF is established, a new question arises: how did it cross the Atlantic Ocean? At the time of the slave trade, the duration of the journey between African and American coasts was longer than 1 month whereas the severe conditions of the crossing certainly did not favor the survival of the patients [35]. Incubation (3 to 6 days) and viremia (less than 10 days before the acquired immunity does not make it disappear) are too short for the virus to have been carried by slaves, if we admit the likelihood that some of them may have been infected at the time of their departure [36, 37]. It is now accepted that the transfer was carried out via the mosquito *Aedes aegypti*, whose eggs survive for several months at desiccation [38], which ensures transovarial transmission of the virus [39–41]. The circulation of the virus and the development of the epidemics for which it is responsible are inseparable from the vectors.

Finally, the search for the pathogen has led to a long debate. Most of the works were performed in the Americas, mainly in Cuba, Brazil and Mexico, at least until the beginning of the twentieth century. This can be explained by the socioeconomic development of the Americas, particularly in the nineteenth century, the demographic changes that this region has experienced and the geopolitical influence it exerted on the world at that time. In addition, the lack of laboratories in Africa before the twentieth century did not favor research on the transmission and etiology of YF.

Brazilian scientists played a leading role. Lacerda isolated a fungus from the viscera and stool of patients [42]. Freire discovered a Cryptococcus that he believed responsible for YF and, after culture and attenuation of the yeast virulence, he experimented to obtain a vaccine [43]. In Rio de Janeiro, Havelburg found a coliform [44], the same year that Sanarelli in Montevideo identified another bacillus from Uruguayan and Brazilian patients [45]. In Cuba, Finlay and Delgado observed a bacterium, which they identified as *Micrococcus tetragenus* Koch and Gaffky 1881, both in the vomit of patients, proboscis of the mosquito and blood from inoculated animals [46]. The microbiologists proceeded by analogy, associating the pathogen with the seasonality of the disease, and inferring its adaptation to the environment in which it develops and/or relating the colors matching those of vomit, liver or skin.

A detailed review of potential candidates as etiological YF agents concluded that it was filterable, i.e., a pathogen not yet called a virus: “It is more than likely that the germs of yellow fever, as well as those of small pox, measles, hydrophobia, etc. belong to a group of organisms, smaller than our bacteria and as yet unknown, awaiting discovery” [47]. The US Mission in Cuba led by Reed has shown that the bacillus isolated by Sanarelli (*Bacilus icteroides*) was not the cause of YF but a secondary contaminant [25]. The French Mission in Brazil confirmed these observations and the filtering property of the YF pathogen [36].

The YF virus was isolated June 30, 1927, from the blood of a Ghanaian patient – who recovered – by Adrian Stokes, who contracted YF and died [48]. The 17D vaccine - still used today - was obtained by attenuation of this strain, called Asibi after the name of the Ghanaian patient, a few years later by Theiler [49], which earned him the Nobel Prize in Medicine. A few months after the discovery by Stokes on December 21, 1927, another strain of the YF virus was isolated at the Institut Pasteur in Dakar [50], from François Miyeli a young patient from Rufisque, Senegal [51]. This strain was at the origin of the vaccine manufactured in Dakar (YF French Neurotropic Vaccine or FNV) [52]. Used throughout French-speaking Africa until 1982, the FNV was discontinued because of its adverse effects [53, 54].

### Epidemiology of yellow fever in Africa

YF has been known in Africa since the end of the fifteenth century through sporadic cases and/or limited small epidemics observed in the colonial counters of the West African coast. In East and South Africa, YF was probably rare or absent from coastal areas because of the lack of an appropriate vector.

**Fig. 5:**
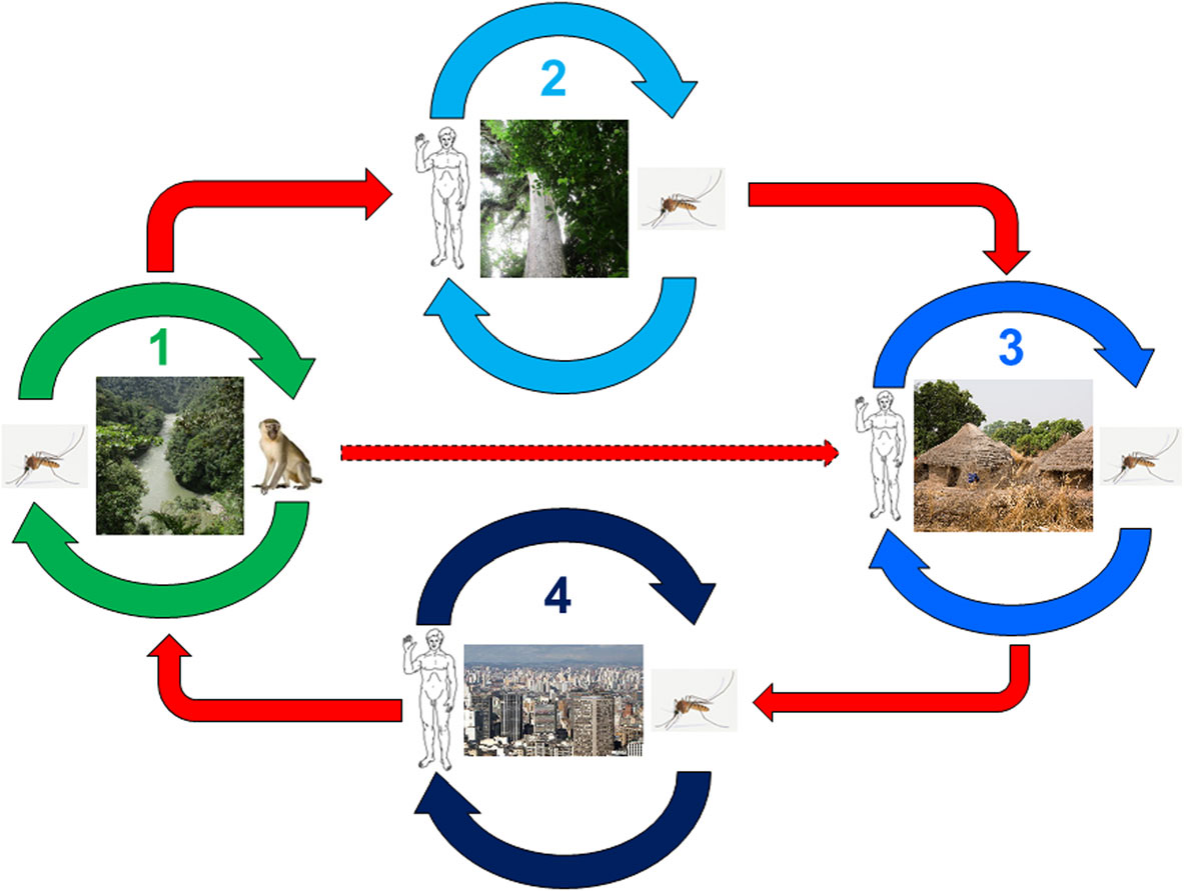
Natural and epidemic cycles of yellow fever: 1 = natural sylvatic cycle; 2 = anthropogenic sylvatic cycle (plantation or deforestation); 3 = village epidemic; 4 = peri-urban or urban epidemic (1 to 4 in Africa, 1 and 4 only in South America) (adapted from [58, 132])

The epidemiology of YF in West and Central Africa was well described in the 1960s to 1980s [55–58] following a long cross-section follow-up of virus circulation during and outside of epidemic periods. It is possible, schematically, to restrict the zones of virus circulation to three regions: a) the main one that harbors the endemic sylvatic cycle, i.e., the natural focus, b) a border zone of emergence, actually a latency area ensuring a transition between the latter and c) the zone of epidemics, visible part of the iceberg, made up of regions inhabited by varying densities of human populations (Fig. 5).

Endemic areas are those where the virus circulates between some *Aedes* species, notably *A. africanus* and *A. furcifer*, and non-human primates, especially arboreal monkeys [56, 57]. In Africa, monkeys are resistant to the YF virus and, if they become infected or sick, do not die but rather become immune [55, 59]. Humans are not present within the endemic areas and may be accidentally infected during a short forest stay, which can lead to sporadic cases and/or limited epidemics leading to the immune protection of the population and restricting the risk of local epidemics. In the zone of emergence, most often in villages bordering forest and savannah or sometimes in extensive plantations, some *Aedes* species, particularly *A. furcifer*, leave the plant coverage, penetrate the inhabited areas and bite humans that they infect [58].

The endemic zone tends to spread, partly because of the transovarial transmission of YF in mosquitos [39–41] and, secondly, because of the anthropization of the environment due to deforestation and population growth. Global warming could play an increasing role in the near future by changing environmental conditions to favor the vectors, in particular through alterations in the rain regime.

In-depth studies of viral infection in wild mammals and specific antibodies showed that vertebrates in general, including monkeys, play only a secondary role in the persistence and resilience of the virus in the sylvatic cycle, because of the short duration of viremia and the resulting definitive immunity, which reflects an effective adaptation of the virus to its transient host [56, 57, 60–62]. Humans are no exception to this rule, whether they live in primary forest like the Pygmies of Central Africa or in villages close to natural foci, although their susceptibility to infection is probably higher [63, 64]. Wild vertebrates, no more than humans, cannot be considered a virus reservoir, a function that has essentially devolved to mosquitoes through the transovarial transmission of the virus. During estivation, the virus is maintained in the eggs of *Aedes*, thereby ensuring infection of the adults at their hatching at the beginning of the following season. On the other hand, monkeys – and possibly other small mammals –, especially the young still immunologically naïve ones, amplify, reveal and propagate the virus from the first rains, thus perpetuating the sylvatic cycle [57].

In the epidemic zone, the virus does not circulate and the wild vector does not occur. The population shows a low immune status, except in immunized individuals, which in case of low immunization coverage explains the intensity of the epidemic [53]. In urban and peri-urban areas, YF transmission is ensured by other *Aedes* species, first of all *A. aegypti*. The virus is spread in the municipality, amplified by the proliferation of *A. aegypti*, where there are favorable conditions, lack of immunity among a large proportion of the inhabitants and, even today, health-system insufficiency based on the tripartite approach: mass vaccination, vector control, screening and treatment of cases placed under bed nets.

YF vaccination was made mandatory in 1941 in all French-controlled colonies. Facilitated by its stability and administration by scarification associated with the smallpox vaccine, mass immunization led to remarkably effective control of YF [5, 53]. Its use was suspended in 1960 in children under 10 years because of the risk of serious adverse effects related to the use of the FNV manufactured in Dakar – although no case had been reported in a dozen years – then definitively interrupted in 1980. The disease reappeared and grew from 1965 in almost all French-speaking countries of sub-Saharan Africa. Since the late 1990s, in the face of renewed outbreaks of YF throughout Africa, sub-Saharan countries are progressively including the more tolerable 17D vaccine in the Expanded Program on Immunization by introducing routine YF vaccination in 9-month-old children together with the measles immunization supported by GAVI (Global Alliance for Vaccines and Immunization).

### Epidemiology of yellow fever in the Americas

In South America, YF was mentioned at the same time as in Africa and in similar circumstances - during the slave trade – first in the Caribbean and in Central America (Yucatan). In Brazil, the first indisputable description of YF was made after the Pernambuco epidemic in Recife and Olinda in 1685 [65]. Legend has it that a boat from the Cape Verde Islands carried slaves with an unknown disease [66]. In fact, the boat had stopped in Guadeloupe, where YF was endemic if not epidemic [27], before reaching the Brazilian coast [67]. It is also possible that Cape Verdean mosquitoes, and not Antillean patients, introduced the virus into Pernambuco. However, the infection of passengers by YF was refuted by Gouy [68] who diagnosed food poisoning by the spoiled meats consumed during the trip based on the kinetics and symptoms of the disease.

Anyway, the penetration of YF in America via Brazil is not excluded because of the considerable economic development of Brazil starting from 1554. The expansion of sugar plantations was favored by the large spaces available and the tax benefits granted by Portugal. On the one hand, the need for labor not met by indigenous natives was assumed by the massive influx of African slaves (3500 individuals per year on average at the end of the sixteenth century). On the other hand, the crossing from Africa to Brazil was shorter than that to the West Indies or North America due to the proximity of both coasts and favorable winds, resulting in the multiplication of YF transport opportunities [35, 69, 70]. In addition, the agricultural techniques favored the development of *A. aegypti* ensuring the perpetuation and dispersal of the virus [69]. YF was reported in the ports of Pernambuco in 1640 and, perhaps, in other regions of Brazil [27, 66]. It is therefore conceivable that the YF virus had been already endemic in Brazil since the sixteenth century and that the epidemic resulted from an indigenous strain emergence.

The endemic zone of YF is located in the Amazon and encompasses, to the west, Peru and Colombia, to the north the Guyana plateau (Venezuela, Guyana, Surinam and French Guiana), and to the northeast, Brazil. The description of the sylvatic cycle was made on the occasion of the great 1942 epidemic in Brazil [71, 72]. The circulation of the virus seems persistent, even if the detailed mechanisms were not yet discovered [73]. It is notable, for example, that specific immunity is maintained in monkeys from many parts of Brazil, even outside the Amazon, regardless of epidemic episodes, suggesting a regular infection of the monkeys by the virus resulting in a lasting immunization in survivors [74]. While the general pattern remains fairly similar to the African one (Fig. 5), there are some important differences. First, the American monkey is susceptible to the YF virus and many individuals die from it [75]. Thus, it not only plays the role of amplifying YF endemia as in Africa [73] but also reveals the risk of epidemics: periodic epizootics that occur near inhabited regions are used as a warning signal [76]. In addition, the vectors are different – at least within the jungle cycle – in which *Haemagogus janthinomys* and *H. leucocelaenus* play an essential role while other species (including *H. capricornii*) or genera, notably *Aedes*, are little involved in the transmission [72, 77–83]. On the other hand, *A. aegypti* and *A. albopictus* represent a major risk of spreading YF in urban and peri-urban areas [84].

Despite common ecological conditions in the Amazonian region, there are two lineages of the YF virus: genotype 1 from Brazil (or possibly from the West Indies) and genotype 2 from Peru [6, 85–87]. A strong genetic heterogeneity of the virus has been developing for some 30 years, increasing since the 2000s, probably as a result of human migrations that play a role of both gene mixing and viral dispersal [85, 88, 89].

The reemergence of YF in Brazil benefits from particularly favorable environmental conditions: high deforestation increasing the contact of human populations with the sylvatic virus, substantial migration between endemic and epidemic regions, poorly controlled urbanization favoring the multiplication of vectors, low immunization coverage in epidemic risk areas [90]. Finally, the role of the recent dengue and Zika epidemics, transmission of which is ensured by the same vectors, in diverting attention and /or complicating the logistics of the health services, remains to be elucidated.

On the occasion of the Rio de Janeiro epidemic of 1900, the French scientific mission demonstrated the vertical transmission of the pathogen in mosquitos, and identified the former as small filtering organism. The first vaccine trials were undertaken [36, 39, 40]. At the end of the mission, new health measures produced spectacular results.

The control of *A. aegypti* started in the 1920s in most Latin American countries, reinforced by the use of DDT from 1947 [91]. Mass vaccination campaigns started after the discovery of the 17D vaccine in 1937. The elimination of the vector and immunization of a large part of the Brazilian population were considered major public health achievements and established an expectation of YF elimination in Brazil and perhaps more widely in Latin America. The persistence of the sylvatic transmission of YF, or “jungle cycle”, and resurgence of *A. aegypti* from jungle foci were unexpected [73, 92].

### The role of the vector and its cooperation with the virus

The YF virus and its vector live in close symbiosis in which the evolution of the former seems irremediably linked to that of the latter. The virus-vector coadaptation is essential for maintaining the sylvatic cycle. This is the case in Africa and South America where the virus adapted to various species of Culicidae in forest areas that have become endemic. Humans become infected and spread the epidemic all the more rapidly so that, on the one hand, the mosquito is both receptive to the virus and anthropophilic, which is the case for *A. aegypti* and *A. albopictus* and, on the other hand, the human population lacks herd immunity.

These two species, in particular *A. aegypti*, favor the transport of the virus because of the resistance of their eggs to the desiccation [93] that leads to the resilience of the YF virus [94, 95]. The spread of YF probably occurred at the same time as that of *A. aegypti*. The short flying distance of *Aedes*, especially *A. aegypti* and *A. albopictus*, leaves no alternative other than passive transport, especially by the transportation of humans or goods [96–102].

The molecular phylogeny of *A. aegypti* strains confirmed the African origin of the species although genetic variability reflected massive mixings due to multiple importations over time from all continents, and human intervention for controlling mosquitoes [103, 104]. In addition, genetic heterogeneity resulted in a high variability in susceptibility to viral infection according to vector strains [105–108]. The original strain – now known as *A. aegypti formosus* as opposed to the subspecies *A. aegypti aegypti*, which is the domestic and urban form of the species – is still present in African jungle where the larvae grow in tree trunks, and whose females are not anthropophilic [109–111]. *A. aegypti formosus* is involved in the transmission of YF in nonhuman primates within the sylvatic cycle [112]. It is not known whether the domestication of *A. aegypti*, which led to its diffusion in America, was earlier or contemporaneous with its transport during the slave trade [111]. In either scenario, *A. aegypti* adaptation to human populations in West Africa was early, which explains the many large urban epidemics in this part of the continent at the end of the eighteenth century [27]. On the other hand, in East Africa, *A. aegypti* does not seem to have adapted to the anthropization of the environment, as evidenced by the scarcity of urban epidemics up to the present day [113].

The colonization of Asia by *A. aegypti* was later, probably at the end of the nineteenth century when it was revealed as an urban vector of dengue fever [114]. Phylogenetic data suggest that the Asian strain originates from the Americas and not from East Africa as might have been expected, probably because of the poor adaptation of the East African strain to humans and its poor susceptibility to the YF virus [33, 108, 111]. In addition, the genetic homogeneity of Asian strains of *A. aegypti* suggests that the introduction was recent and accomplished by a limited number of entries [115]. However, although the current strain of *A. aegypti aegypti* is monophyletic [110], it has important local polymorphisms [116, 117] that could explain, at least in part, the variability of the vector competence [32, 105, 107, 118].

### Absence of the YF from Asia and Oceania

The absence of YF from Asia and Oceania remains an enigma. The population is susceptible to the virus as shown by Asian patients living in endemic areas (Africa or South America). *A. aegypti*, although a recent arrival, is present in many places in Asia and Oceania. In addition, the abundant circulation of other arboviruses, dengue, chikungunya and Zika, the latter two coming from Africa like YF, indicates the possibility of YF extension in Asia. Recently, several human cases of YF introduced from Angola have been diagnosed in China [119], increasing YF outbreak fears. Several studies attempted to explain the absence of YF in Asia. The following main reasons were given: a) poor competency of the vector for the virus [32, 105, 108, 118], b) poor adaptation of the vector to humans and competition between vectors (Abrão and Fonseca, 2006, quoted in [120]), c) competition between Flavoviridae in the vector at the expense of YF and d) cross-reactivity with other Flavoviridae, including dengue fever, in humans and other animals [121, 122]. Recent modeling of YF risk in Asia showed that two major factors limited the probability of YF expansion in Asia (“Asian hypothesis”) and dengue fever in Africa (“African hypothesis”) [123]. The first was based on cross-immunity between Flavoviridae – especially between dengue and YF, which could limit the risk of double infection – and high viremia and severe clinical forms of YF [121, 122]. The second was based on the competition between *A. aegypti* and *A. albopictus* as well as on their respective competencies in the transmission of dengue and YF to explain the absence of the latter in Asia and the rarity of the former in Africa [120]. Another explanation was the low probability of transporting the virus from Africa – or the Americas – to Asia and the Pacific. The slave trade to Asia was much smaller than the trade to the Americas. This reduces the probability – as to the transport of the virus into the Americas before air transport – by infected humans [124]. Mosquito transport did not occur, either from Africa or America, for reasons that have not yet been elucidated.

### Rooting out and controlling of yellow fever in Brazil

Since its introduction in the seventeenth century, YF remained in the Amazonian region in the form of a jungle cycle that causes rural, peri-urban or urban epidemics at regular intervals. The sylvatic cycle is maintained by wild endemic vectors present before the introduction of YF in Brazil – belonging to the genera *Haemagogus* and *Sabethes* – playing the role of virus reservoir, especially in the Amazon that is the refuge zone of the virus [3, 79–83]. Extension to peri-urban and urban areas involves *A. albopictus* and *A. aegypti* respectively, and follows the routes of human migrations, particularly forest galleries [4].

Clinically, the Brazilian YF does not differ from the forms described in Africa or other American countries. The high case fatality rate (up to 50%) is likely not to be the manifestation of a particular virulence but resulted from underreporting of benign cases [125].

**Fig. 6:**
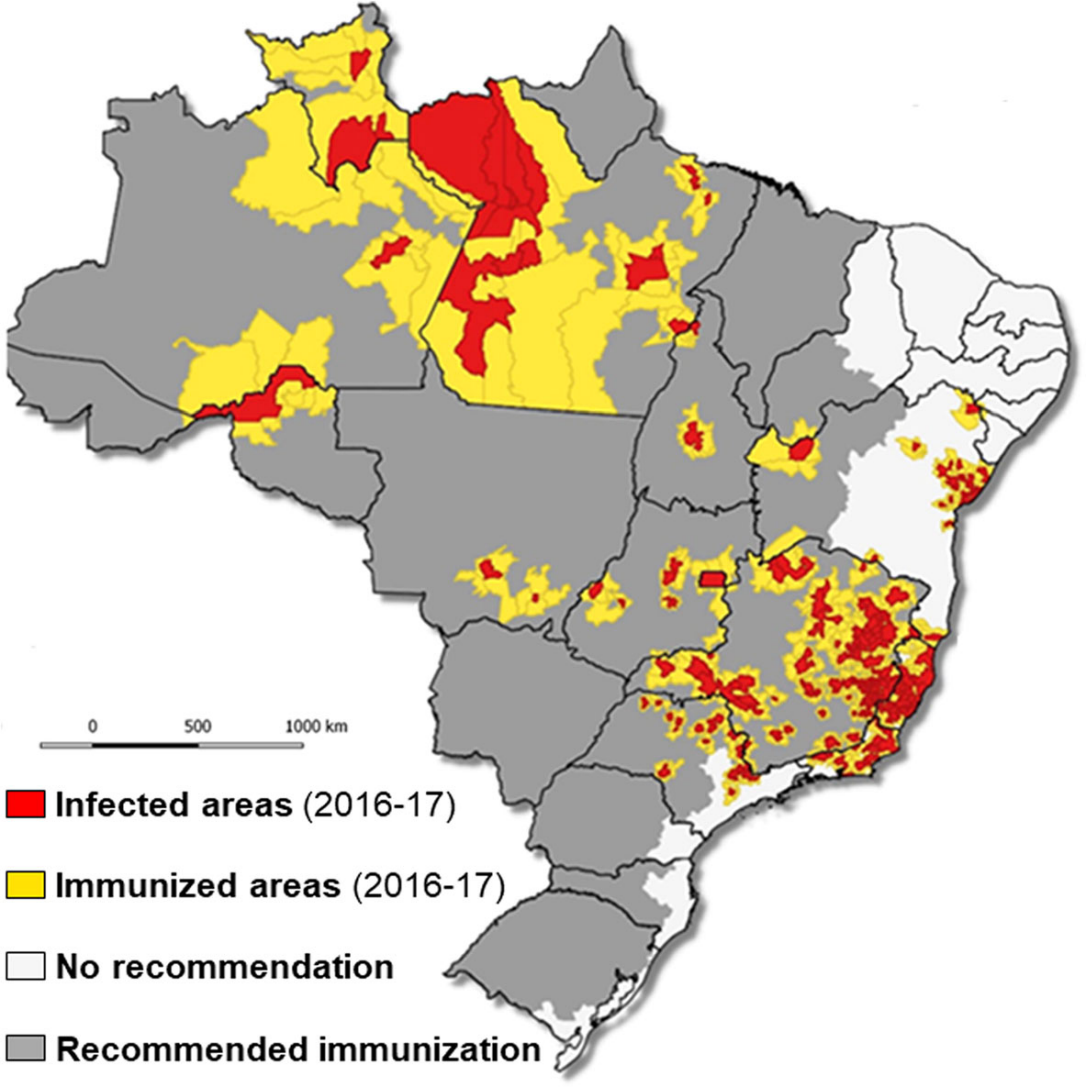
Municipalities with human cases and non-human primates: infected and immunized areas, Brazil, 2016/2017 [2]

The vector control carried out at the beginning of the twentieth century during the Rio de Janeiro epidemic was organized nationwide in 1928–29. It was reinforced by YF immunization in 1937 and DDT sprayings initiated 10 years later, which significantly reduced the burden of YF, that was considered as having been eliminated from Brazil in the 60s [91, 126]. However, the epidemics of dengue in 1963–64 then in 1968–69 marked the beginning of the re-infestation of the continent by *A. aegypti*, confirmed between 1971 and 1999 [127], which resulted in the return of YF and, more recently, the installation of other arboviruses (chikungunya and Zika, in particular) [2]. Several factors can explain this phenomenon. The most important is, almost certainly the reinvasion of *A. aegypti* from isolated and poorly controlled Amazonian and Southern Brazilian forest localities, as a result of a relaxation of vector control and vaccination campaigns, as well as the deterioration of health systems in some remote areas [92, 103, 128]. Another reason could be the insufficiency of vaccination coverage, which should have favored the extension of the disease beyond its natural limits, particularly in the regions of Brazil where vaccination was not recommended, e.g., in northeast and southern Brazil [90, 129]. Nevertheless, YF now seems well established in the form of a deeply rooted jungle cycle, as shown by the numerous epizootics reported in recent years (Fig. 6). Finally, the sudden population growth and unplanned urbanization that results in substandard housing, and inadequate water supply and waste management systems, at least partially explain the failure of preventive measures and the delay in the management of cases [85, 128]. The vectorial competence of *A. aegypti* and *A. albopictus* reinforce the risk of YF extension in cities, especially in the overcrowded suburbs of major Brazilian cities [3, 84].

The attempt to eliminate the YF during the twenty-first century, despite a relative failure, probably contained the risk of urban and peri-urban epidemics. For more than 50 years, Brazilian YF epidemics – and the most recent one was no exception – were sylvatic and involved wild vectors (*Haemagogus* and *Sabethes*, as well as non-anthropophilic *Aedes*, i.e. other species than *A. aegypti* and *A. albopictus*) and spared densely populated areas. So far, humans have been infected outside cities and their suburbs from multiple sylvatic foci [73]. However, the sylvatic cycle is expanding, and getting closer to the cities, infesting forest areas that could even be included in the conurbations of southern Brazil, notably in the states of Rio de Janeiro, São Paulo and Rio Grande do Sul, highlighting the risk of metropolitan epidemics. [80–84, 130, 131]. The epidemiological surveillance should be performed at two levels. On the one hand, the sentinel role of the monkeys that are the first to express the reemergence of the YF, dying from the disease, must be favored [76]. On the other hand, the clinical diagnosis of YF index cases, which may reveal an increased risk of epidemic, must be reinforced. The “syndromic surveillance” approach recommended by US Centers for Disease Control and Prevention increases the sensitivity – and specificity – of first-line clinical detection [132]. This method speeds up the diagnostic process, which is employed to organize case management and contain the epidemic.

The return of YF has led to a considerable increase in the genetic diversity of its South American genotype I, leading to the appearance of a new viral line [89]. The reasons for this diversification and its consequences, particularly as to transmissibility and virulence, remain to be clarified. One of the pending issues is the need to assess the capacity of the YF virus currently present in Brazil to invade peri-urban and urban environments spread by the *A. aegypti* and *A. albopictus* populations that abound [3, 4].

Two strategies – certainly not exclusive – enable the containment of YF: vector control and immunization of populations at risk. The former is very effective but expensive. It requires a constant effort wherever the risk of vector development is high. The recent epidemics of dengue, chikungunya and Zika in Brazil demonstrated the limits of this strategy [133]. However, integrated control – taking into account sanitation – and mutualized with other vector-borne diseases, is cost-effective and should be favored [134, 135]. Vaccination against YF is based on a selective strategy – routine mandatory vaccinations in endemic areas of Brazil, voluntary vaccinations elsewhere such as the east and south of the country, and large-scale vaccinations of populations threatened by an epidemic. In Brazil, vaccine coverage is irregular, with a drop in coverage between 1990 and 2010, and insufficient in States where it is not compulsory [136], which requires making a considerable effort to catch up on vaccinations in the event of an epidemic. This defective coverage results from recurrent vaccine shortages and poor tolerance [137–139].

Once the causative agent of the YF was identified in 1927 [48], the vaccine was discovered 10 years later [49]. It is a live virus attenuated by repeated passages into chicken embryos (YF 17D), and genetically stable. It replicates in the vaccinated individuals and confers protection for life [34, 138, 139]. The effectiveness of the vaccine in controlling YF has been emphasized above in relation to its epidemiological description of the in both Africa and the Americas. In the current Brazilian context, vaccination of at-risk populations, including those living outside endemic areas, appears to be a strategy of choice provided that at least 80% of the resident population is covered to obtain an effective herd immunity [139]. However, global production – about 80 million doses annually – is insufficient in some years to cover the needs, i.e. routine newborn vaccination, immunization of travelers and protection of populations at risk of epidemic [54, 138]. The reduction of the vaccine dose administered – up to one-fifth of the recommended dose – conferred sufficient immunity and would allow, in the event of an epidemic, to respond to the needs, although these results require confirmation in real-life situations [54, 139–142].

Another important issue is the incidence of serious adverse events (SAE). In addition to vaccine hypersensitivity and anaphylactic shock, SAEs were categorized into two groups: YFV-associated neurotropic disease (YFV-AND), including encephalitis, myelitis, acute disseminated encephalo-myelitis, and Guillain-Barré syndrome, and YF-vaccine-associated viscerotropic disease (YDV-AVD) responsible for multi-organ failure [139]. Risk factors are age (young children under 6 months and adults over 50), pregnancy and immunodeficiency. Most SAEs concern primary vaccination [143, 144]. Based on meta-analyses in many countries with reliable pharmacovigilance, the incidence of SAEs was estimated to be 11.1–15.6 per million vaccinated persons, including 6.6 YFV-AND and YFV –AVD cumulated, with wide variations depending on the country [145]. These are linked both to the surveillance system and to the recruitment of vaccinated persons: in YF-free regions, the latter are most often travelers whose age is on average significantly higher than people from endemic countries where routine immunization targets young children. Despite the high incidence of SAEs, the risk-benefit profile remains extremely favorable for vaccination in endemic areas [139]. The mortality due to SAEs is below one per million vaccinated people [145], which must be compared with the 33% case fatality rate of YF in Brazil (Table 1). In addition, it is likely that the risk of SAEs increases during large-scale vaccination campaigns due to reduced attention to risk factors in vaccinated people and conditions of storage and vaccine administration. The vaccinations carried out in Brazil and Argentina during the recent YF outbreak confirmed these data, showing an SAEs incidence ranging from 8.3 to 12.4 per million respectively, mainly involving people at risk, especially those over 50 years old [137, 146]. Considering the risks of urban epidemics and incidence of SAEs, it is urgent to develop a more tolerable vaccine. The vaccine strategy will need to be reconsidered to extend vaccine coverage to the whole country, including major urban metropolises.

## Conclusion

The physiognomy of YF has reemerged abruptly over the last 50 years while the disease was likely to be controlled. Consequently, it is no longer a question of its eradication, if only because of the sylvatic foci present on two continents, but rather of limiting the epidemic risk [147].

The reservoir of YF, both in Africa and the Americas, is the mosquito, especially the sylvatic species that maintain the jungle cycle. The short viremia and definitive immunity – or death – that follow the viral infection in mammals do not allow their consideration as a proper virus reservoir. However, they ensure amplification of the latter and, in the Americas in particular, are an alarm signal of viral reemergence that can result in human outbreaks able to extend into urban areas.

The history of YF, especially its installation in the Americas five centuries ago, taught us that the virus and its vector presented a remarkable capacity for environmental adaptation. The pair seem to react very quickly to the conditions that they face. While until 1960, the virus was spread by boat – limited to transport through mosquitos – which required time and allowed the implementation of preventive or remedial measures, it is now rapidly spread via aircraft carrying infected humans, a scenario that is more difficult to control.

However, there are flaws in the transmission of the YF, as evidenced by its absence from Asia. The emergence or re-emergence of vector-borne diseases depends on complex, entangled biological, climatic, ecological, socio-economic and political factors [148]. Three conditions are necessary to produce YF epidemics: a) the introduction of the virus into a non-immune human community, b) presence of competent and anthropophilic vectors and, c) insufficiency of prevention and/or adequate management. On the other hand, there are two weapons available to fight YF: vector control and immunization of human populations [147]. The first has the advantage of allowing the control of many other parasitic and viral diseases transmitted by arthropods, and may result in the mutualization of resources. It involves financial and ecological constraints that can limit its application and effectiveness. Immunization is both cheaper and very effective, subject to strict and permanent application.

The objectives of the WHO are therefore to protect the at-risk population, prevent international spread and rapidly contain epidemics. These objectives include a prevention component through appropriate urban development and an immediate response in the event of an YF outbreak by combining vector control against *Aedes* larvae and adults, and preventive mass immunization [149]. In Brazil, preventive vaccination is underway. However, it is necessary to reconsider the vaccination schedule. While waiting for a safer YF vaccine, vector control measures and epidemiological surveillance should be strengthened to initiate emergency large-scale vaccination campaigns along two lines: monkey mortality [76, 150] and syndromic surveillance in sentinel hospitals [132].

Public health strategies must combine both scientific and political criteria. The history of YF stressed, as many authors have already noted, that the opinions of scientists – sometimes giving rise to expertise followed by the authorities – often came from assertions based on subjective arguments rather than evidence based on observations validated experimentally. Controversies conceal ideological cleavages, even political ones, which divert the public debate from its objectives of prevention and management, make them lose their effectiveness, and involve useless and considerable expenses [15, 16].
